# Primary malignancy in giant cell tumor: a case report

**DOI:** 10.1590/S1516-31802009000500011

**Published:** 2010-02-03

**Authors:** Rosalvo Zósimo Bispo, Olavo Pires de Camargo, Cristiane Marie Ida, André Mathias Baptista, Marcelo Barbosa Ribeiro, José Marcello Bruno, Cláudia Regina Gomes Cardim Mendes de Oliveira

**Affiliations:** I MD, MSc. Traumatologist and orthopedist. PhD student, Department of Orthopedics and Traumatology, Faculdade de Medicina da Universidade de São Paulo (FMUSP), São Paulo, Brazil.; II MD, PhD. Titular professor and Head, Department of Orthopedics and Traumatology, Faculdade de Medicina da Universidade de São Paulo (FMUSP), São Paulo, Brazil.; III MD. Pathologist, Division of Anatomical Pathology, Faculdade de Medicina da Universidade de São Paulo (FMUSP), São Paulo, Brazil.; IV MD, MSc. Traumatologist and orthopedist. Attending physician, Department of Orthopedics and Traumatology, Faculdade de Medicina da Universidade de São Paulo (FMUSP), São Paulo, Brazil.; V MD. Traumatologist and orthopedist. Master’s degree student, Department of Orthopedics and Traumatology, Faculdade de Medicina da Universidade de São Paulo (FMUSP), São Paulo, Brazil.; VI MD. Traumatologist and orthopedist. Postgraduate student, Department of Orthopedics and Traumatology, Faculdade de Medicina da Universidade de São Paulo (FMUSP), São Paulo, Brazil.; VII MD, PhD. Pathologist and Head of the Division of Anatomical Pathology, Faculdade de Medicina da Universidade de São Paulo (FMUSP), São Paulo, Brazil.

**Keywords:** Bone neoplasms, Bone tissue, Giant cell tumor of bone, Osteosarcoma, Histiocytoma, malignant fibrous, Neoplasias ósseas, Osso e ossos, Tumor de células gigantes do osso, Osteossarcoma, Histiocitoma fibroso maligno

## Abstract

**CONTEXT:**

Primary malignancy in giant cell tumor (PMGCT) is rare. It is defined as a high-grade sarcoma originating in a giant cell tumor (GCT) and seems to behave less aggressively than its secondary counterpart does.

**CASE REPORT:**

This report presents the case of a 39-year-old female with pain in her left shoulder for one month. Radiography showed a pathological fracture of the proximal humerus associated with an osteolytic lesion. Histopathological analysis showed typical areas of GCT juxtaposed with a sarcomatous component.

**CONCLUSIONS:**

PMGCT seems to behave less aggressively than secondary malignancy in GCT, and it may simulate its more common benign counterpart clinically and radiographically. However, it requires a more aggressive type of treatment.

## RESUMO

**CONTEXTO:**

Malignização primária no tumor de células gigantes (MPTCG) é rara. Ela é definida como um sarcoma de alto grau originário de um tumor de células gigantes que parece ser menos agressivo que o tipo secundário.

**RELATO DE CASO:**

Relatamos um caso de uma paciente de 39 anos de idade, com dor no ombro esquerdo há um mês. A radiografia mostrou uma fratura patológica do úmero proximal associada a uma lesão osteolítica. O exame histopatológico revelou típicas áreas de tumor de células gigantes justapostas por um componente sarcomatoso.

**CONCLUSÃO:**

MPTCG parece se comportar menos agressivamente que a neoplasia secundária do tumor de células gigantes (TCG), e pode simular o TCG, que é mais comum, tanto clínica como radiograficamente. Entretanto, exige tratamento mais agressivo.

## INTRODUCTION

Giant cell tumor (GCT) is a primary locally aggressive bone neoplasm characterized by stromal mononucleated cells associated with uniformly distributed osteoclast-like giant multinucleated cells. This tumor type accounts for 4-5% of all primary bone tumors and 20% of benign bone tumors.^1^ Skeletally mature patients ranging from 20 to 45 years of age, especially women, are affected. GCT usually involves the epiphyseal or meta-epiphyseal region of long bones, particularly the femur and tibia, and appears radiographically as a purely osteolytic eccentric lesion.^2^ Malignancy in GCT (MGCT) cases is defined as high-grade sarcomas originating in a GCT (primary) or at the location of a previous well-documented GCT (secondary).^1^ Primary malignancy in GCT (PMGCT) is the rarest type, and it seems to behave less aggressively than the secondary type does.^3^^,^^4^

Here, we report the clinicopathological features of a rare case of PMGCT that was treated by the Orthopedic Oncology Group at a reference service, and we briefly review the pertinent literature.

## CASE REPORT

**Figure 1.**
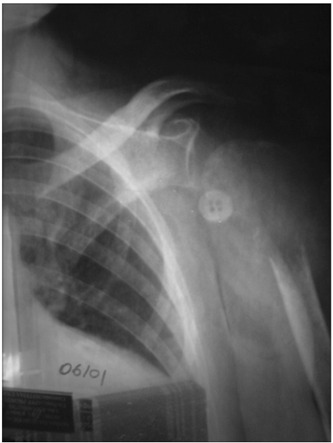
Radiograph of the left shoulder showing a pathological fracture of the proximal humerus. Note an osteolytic lesion located in the meta epiphyseal region of the bone.

A 39-year-old female presented with pain in her left shoulder for one month. Clinical examination revealed slight swelling and tenderness of the proximal part of the left upper limb, with unremarkable neurological examination. No calcium metabolism abnormality was identified. Plain X-rays showed a pathological fracture of the proximal humerus associated with an osteolytic lesion (Figure 1). Scintigraphic evaluation revealed a lesion with high uptake. An incisional biopsy was performed and the diagnosis of GCT was made.

**Figure 2.**
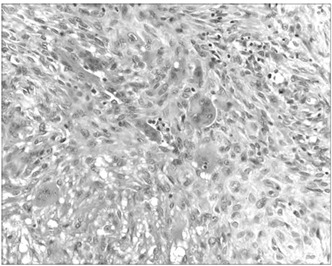
Photomicrograph showing typical areas of giant cell tumor characterized by stromal mononucleated cells associated with uniformly distributed osteoclast-like giant multinucleated cells (200 x, hematoxylin and eosin, HE).

**Figure 3.**
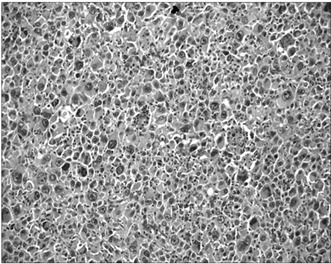
Photomicrograph showing a juxtaposed sarcomatous component (200 x, hematoxylin and eosin, HE).

Microscopic examination showed typical areas of GCT characterized by stromal mononucleated cells, which were associated with uniformly distributed osteoclast-like giant multinucleated cells (Figure 2), in which the nuclei of the stromal cells were identical to the nuclei of the giant cells. Juxtaposed to these areas, there was a sarcomatous component composed of anaplastic spindle cells showing pleomorphism, nuclear hyperchromasia, increased nuclear/cytoplasmic ratio and atypical (tri-multipolar) mitotic figures (Figure 3). There was predominance of atypical stromal spindle cells over the giant cells, as well as osteoid matrix synthesis by these atypical spindle cells (osteogenic sarcoma/osteosarcoma), thereby giving the diagnosis of PMGCT of the “dedifferentiated” type. Foci of coagulative necrosis were observed as well. The mitotic index was up to 23 mitoses/10 high-power fields (HPF). Significant areas of the lesion were represented by the sarcomatous component (70-80%). Vascular emboli were also observed.

**Figure 4.**
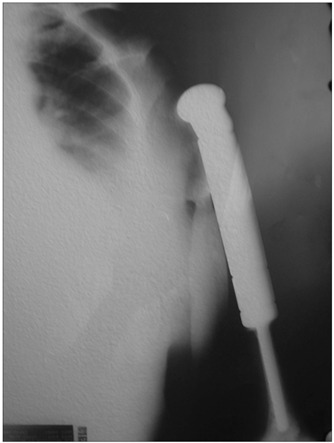
Radiograph showing wide resection of the proximal humerus with endoprosthesis replacement.

Wide resection was performed, with reconstruction using an endoprosthesis (Figure 4). After the operation, adjuvant chemotherapy based on the protocol used for osteosarcoma was provided. After four years of follow-up, the patient has no clinical or radiological evidence of recurrence.

## DISCUSSION

Primary malignancy in GCT (PMGCT) is rare and represents less than 1% of GCT.^1^^,^^3^^,^^4^^,^^5^ Likewise, the case reported here accounted for 0.22% of 435 cases treated in our service over a 53-year-period This case was accepted as PMGCT since it showed typical areas of GCT (osteoclast-like giant multinucleated cells uniformly distributed among stromal mononucleated cells with nuclei similar to the nuclei of the giant cells) juxtaposed with areas of high-grade sarcoma represented by an osteogenic sarcoma (osteosarcoma).

Recently, the term “malignancy in giant cell tumor” has been preferred for GCT cases that show sarcomatous areas synchronously (primary) or that are replaced by a malignant component metachronously (secondary).^6^ “Malignant giant cell tumor” is a non-specific term used in the past for different neoplasms with giant cells, such as “giant cell-rich” osteosarcoma, malignant fibrous histiocytoma with giant cells and GCT with different degrees of anaplasia or with metastasis.^3^

The histopathological classification criteria for the sarcomatous component in PMGCT are not well established in the literature. Originally, Jaffe^7^ described morphology in which the stromal mononucleated cells showed noticeable atypia (Grade III). Subsequently, Nascimento et al.^4^ proposed that there should be significant areas of high-grade non-osteogenic sarcoma, thereby avoiding misdiagnosis of “giant cell-rich” osteosarcomas as PMGCT. Recently, not only has it been possible for the sarcomatous component to be either osteogenic (osteosarcoma) or non-osteogenic (malignant fibrous histiocytoma/fibrosarcoma), but also, analogous to the dedifferentiation process of tumor progression seen in liposarcomas,^8^ chondrosarcomas,^9^ chordomas^10^ and parosteal osteosarcomas,^11^ the expression “dedifferentiated giant cell tumor” has been applied to PMGCT cases in which the malignant component is not represented by areas morphologically reminiscent of a GCT (Jaffe and Lichtenstein’s grade III GCT).^5^^,^^12^

The diagnosis of PMGCT may be extremely difficult.^1^ Pleomorphism and some degree of cell atypia, which is considered degenerative in nature,^13^ as well as areas of coagulative necrosis, vascular emboli and metastasis,^6^ are not considered to be criteria for malignancy.^1^ The differential diagnosis with “giant cell-rich” or “osteoclast-rich” osteosarcoma in a tumor in which the cells synthesize osteoid and are associated with osteoclast-like giant multinucleated cells may be achieved through the absence of typical GCT areas and through a diaphyseal or meta-diaphyseal location.^14^ On the other hand, malignant fibrous histiocytomas with giant cells are characterized by a storiform pattern without a GCT component.

Although the histogenesis of GCT is still unknown, there is speculation about it. Brien et al.^15^ suggested a hypothetic model with three cell types: mesenchymal, spindle, and mononucleated and multinucleated histiocytic types. Initially, there would be proliferation of the mesenchymal component with production of high levels of osteoclast stimulator/activator factors. In response, the histiocytic cells would fuse into the osteoclast-like multinucleated giant cells. Rarely, the primitive mesenchymal component would dedifferentiate and originate a high-grade sarcoma (osteo/fibrosarcoma, or malignant fibrous histiocytoma), thus characterizing PMGCT. This phenomenon might be time-dependent,^12^^,^^16^ or even associated with degenerative cellular events, since PMGCT affects patients one^1^^,^^2^ to three decades^3^^,^^4^ later.

Similarly to GCT, PMGCT presents as a painful lesion that typically involves the ends of the long bones, in a way that might be impossible to differentiate it clinical and radiographically from an ordinary GCT.^12^ The differential diagnosis is essential, since GCT is a potentially curable neoplasm that carries five-year overall survival of about 90%.^17^^,^^18^ Nonetheless, it has locally aggressive behavior, since the recurrence rates range from 6.3 to 33%^18^^,^^19^^,^^20^ depending on the treatment,^21^ and it might even be fatal.^2^ Primary malignancy in GCT, however, is a sarcoma that seems to behave less aggressively than the secondary type of MGCT, especially when the latter is related to prior radiotherapy.^2^^,^^3^^,^^4^

No mainstay therapy has yet been established, since this is an extremely rare entity. Wide surgical resection has been recommended. There is some evidence that surgery associated with chemotherapy is more efficient than surgical resection alone.^12^

## CONCLUSION

Primary malignancy in giant cell tumor seems to behave less aggressively than secondary malignancy in giant cell tumor, as has been reported in the literature. It is essential to recognize this rare entity microscopically, since it may simulate its more common benign counterpart clinical and radiologically. However, differently, it requires a more aggressive type of treatment.
